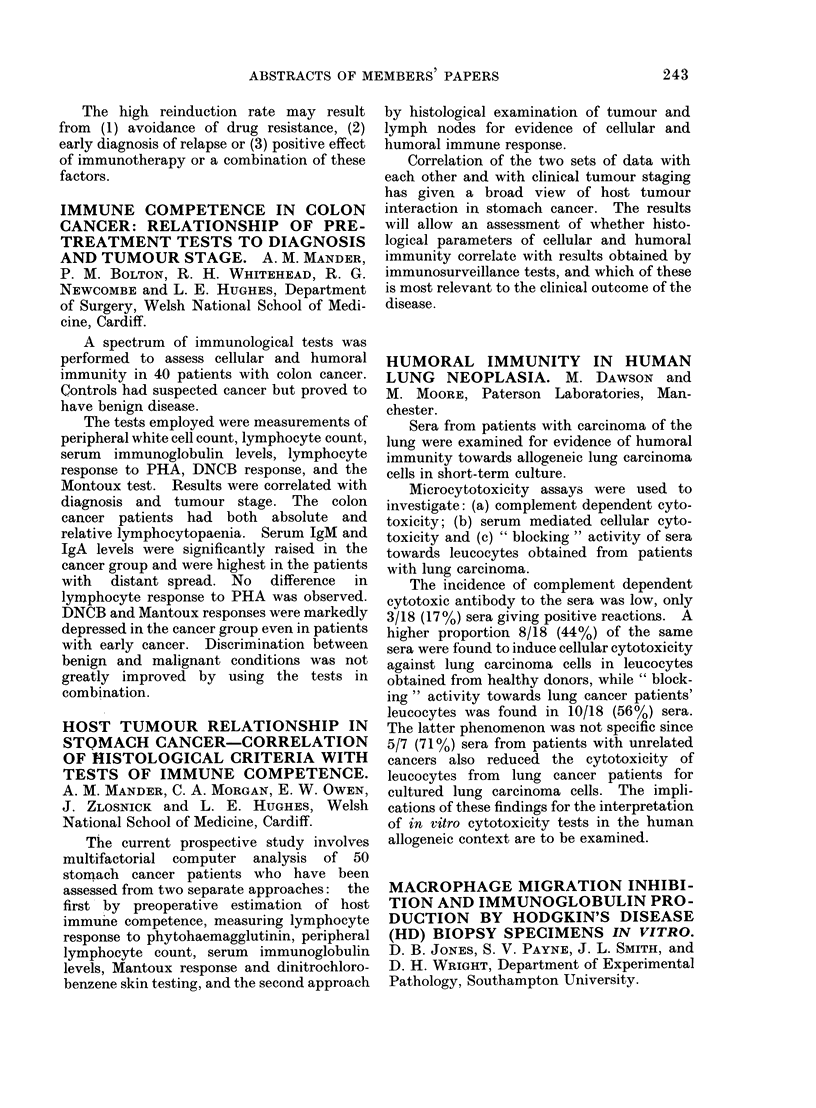# Proceedings: Host tumour relationship in stomach cancer--correlation of histological criteria with tests of immune competence.

**Published:** 1975-08

**Authors:** A. M. Mander, C. A. Morgan, E. W. Owen, J. Zlosnick, L. E. Hughes


**HOST TUMOUR RELATIONSHIP IN STOMACH CANCER—CORRELATION OF HISTOLOGICAL CRITERIA WITH TESTS OF IMMUNE COMPETENCE.** A. M. MANDER, C. A. MORGAN, E. W. OWEN, J. ZLOSNICK and L. E. HUGHES, Welsh National School of Medicine, Cardiff.

The current prospective study involves multifactorial computer analysis of 50 stomach cancer patients who have been assessed from two separate approaches: the first by preoperative estimation of host immune competence, measuring lymphocyte response to phytohaemagglutinin, peripheral lymphocyte count, serum immunoglobulin levels, Mantoux response and dinitrochlorobenzene skin testing, and the second approach by histological examination of tumour and lymph nodes for evidence of cellular and humoral immune response.

Correlation of the two sets of data with each other and with clinical tumour staging has given a broad view of host tumour interaction in stomach cancer. The results will allow an assessment of whether histological parameters of cellular and humoral immunity correlate with results obtained by immunosurveillance tests, and which of these is most relevant to the clinical outcome of the disease.